# Role of adjunctive treatment strategies in COVID-19 and a review of international and national clinical guidelines

**DOI:** 10.1186/s40779-020-00251-x

**Published:** 2020-05-05

**Authors:** Xinni Xu, Yew Kwang Ong, De Yun Wang

**Affiliations:** 1 Department of Otolaryngology-Head and Neck Surgery, National University Hospital System, Singapore, Singapore; 2 Department of Otolaryngology, Yong Loo Lin School of Medicine, National University of Singapore, Singapore, Singapore

**Keywords:** COVID-19, Adjunctive treatment, Chloroquine, Lopinavir-ritonavir, Remdesivir, Corticosteroids, Umifenovir, Convalescent plasma

## Abstract

The coronavirus disease (COVID-19) pandemic has led to a global struggle to cope with the sheer numbers of infected persons, many of whom require intensive care support or eventually succumb to the illness. The outbreak is managed by a combination of disease containment via public health measures and supportive care for those who are affected. To date, there is no specific anti-COVID-19 treatment. However, the urgency to identify treatments that could turn the tide has led to the emergence of several investigational drugs as potential candidates to improve outcome, especially in the severe to critically ill. While many of these adjunctive drugs are being investigated in clinical trials, professional bodies have attempted to clarify the setting where the use of these drugs may be considered as off-label or compassionate use. This review summarizes the clinical evidence of investigational adjunctive treatments used in COVID-19 patients as well as the recommendations of their use from guidelines issued by international and national organizations in healthcare.

## Background

The current millennia has witnessed the emergence of three coronaviruses of epidemic proportions: the severe acute respiratory syndrome coronavirus (SARS-CoV), Middle East respiratory syndrome coronavirus (MERS-CoV) and most recently, the severe acute respiratory syndrome coronavirus 2 (SARS-CoV-2) which is responsible for the coronavirus disease 2019 (COVID-19). COVID-19 has proven to be the most pervasive of the three, far outstripping its predecessors in terms of sheer numbers infected and lives claimed.

The global impact of the outbreak has led to a race to develop vaccines and identify potential cures. However vaccines are realistically a long way from becoming publicly available, even though some have already accelerated towards human trials [1]. In the meantime, investigational therapies are being explored as potential adjuncts to standard supportive care [2]. These are multi-pronged approaches directed towards viral inhibition, suppression of the secondary effects of cytokine storm and / or modulation of the host immune system to mount its defenses.

The World Health Organization (WHO) states there is currently no evidence for any specific anti-COVID-19 treatment [3]. Beyond the standard of care, it recommends that investigational therapies for COVID-19 should only be used in approved, randomized, controlled trials. Whilst the medical profession awaits the results of large scale, well-designed clinical trials that are already ongoing, several smaller studies have emerged with early evidence where adjunctive treatments might improve clinical outcome. Some national professional bodies have put together guidelines on treatment of COVID-19 based on clinical experience, published evidence and/or expert consensus. The objectives of this article are to review the clinical evidences of these investigational treatments used in COVID-19 patients and summarize some of the clinical guidelines on the use of these drugs. The management of concurrent infection, sepsis, shock, haemodynamic compromise, respiratory failure or acute respiratory distress syndrome (ARDS) will not be covered as it is considered part of standard care. This article is intended to critically appraise the evidence, rather than endorse the use of these empiric drugs. We hope that it provides some clarity to the treatment options of these patients amidst the trove of information in the literature.

## Identification of clinical studies

A literature search was conducted in PubMed and Cochrane Library to identify published studies examining investigational drugs used to treat COVID-19. The keywords “COVID-19”, “SARS-CoV-2”, and “2019 novel coronavirus” were used in the search strategy. The systematic searches for therapeutic drugs were carried out independently by all authors using the key words “drug”, “therapeutic”, “treatment”, “therapy” and “guidelines”. References of all identified studies were examined to ensure that all relevant studies were collected. Individual case reports were included due to the small number of articles fulfilling the inclusion criteria, but were used primarily to examine for reports on adverse effects. The findings of all included studies were summarized in a standardized table, and the quality of each study was evaluated based on the Oxford levels of evidence from level 1a to 5 (Table 1) [34].

**Table 1:** Summary of clinical studies on investigational therapies in COVID-19 patients

Study	Study location	Study design	Study groups	Clinical endpoint	Adverse effects	Conclusions	LOE
Chloroquine/ Hydroxychloroquine (HCQ)							
Gautret [4]	Marseille, Nice, Avignon, Briançon, France	Prospective cohort study (*n* = 42)	1. HCQ (200 mg tid for 10 days) + azithromycin (500 mg on day 1, followed by 250 mg od for 4 days) (*n* = 6) 2. HCQ (200 mg tid for 10 days) (*n* = 14) 2. Controls (*n* = 16)	1. Virological clearance at day 6 post-inclusion. 2. Virological clearance over time. 3. Clinical follow-up. 4. Side effects.	Not reported	HCQ improved rate of viral clearance. Its effect appeared enhanced by azithromycin.	2b
^a^Chen [5]	Wuhan, China	RCT (*n* = 62)	1. HCQ (200 mg bid for 5 days) (*n* = 31) 2. No HCQ	1. Time to clinical recovery 2. Clinical characteristics and radiologic results 5 days after treatment 3. Severe adverse reactions	Mild: rash, headache	HCQ shortened time to clinical recovery and hastened improvement in pneumonia	2b
Chen [6]	Shanghai, China	RCT (*n* = 30)	1. HCQ (400 mg/d for 5 days) (*n* = 15) 2. Controls (*n* = 15)	Negative conversion rate of viral nuclei acid in pharyngeal swab on day 7 of treatment	Diarrhoea, elevated aspartate aminotransferase, disease progression	No clear benefit in common COVID-19	2b
^a^Magagnoli [7]	South Carolina, Virginia, USA	Retrospective cohort study (*n* = 368)	1. HCQ (*n* = 97) 2. HCQ + azithromycin (*n* = 113) 3. No HCQ (*n* = 158) (doses and duration unknown)	1. Result of hospitalisation (discharge or death) 2. Need for ventilation 3. Result of hospitalisation among patients requiring ventilation	Not reported	Risk of death from any cause higher in the HCQ group. HCQ with or without azithromycin did not reduce risk of ventilation	2b
^a^Borba [8]	Manaus, Brazil	Double-blinded, randomized phase IIb clinical trial (*n* = 81)	1. High dose chloroquine (600 mg bid for 10 days) + ceftriaxone (1 g bid for 7 days) + azithromycin (500 mg od for 5 days) (*n* = 41) 2. Low dose chloroquine (450 mg bid on day 1, then od on days 2–5) + ceftriaxone (above dose) + azithromycin (above dose) (*n* = 40)	Safety and efficacy of chloroquine at high and low doses	Severe rhabdomyolysis (1 patient), prolonged QTc especially in high dose group at days 2 & 3, ventricular tachycardia followed by death (2 patients)	High dose chloroquine should not be recommended due to safety concerns. Recruitment of patients to high dose arm prematurely halted.	2c
Molina [9]	Paris, France	Prospective case series (*n* = 11)	HCQ (600 mg/day for 10 days) + azithromycin (500 mg on day 1, followed by 250 mg od for 4 days)	Nil	Prolonged QT interval resulting in discontinuation of HCQ (1 patient)	No clear evidence of antiviral or clinical benefit of HCQ + azithromycin in severe COVID-19	4
^a^Mahévas [10]	Paris, France	Cohort study (*n* = 181)	1. HCQ (600 mg/d) (*n* = 84) 2. No HCQ (*n* = 97)	1. Transfer to ICU within 7 days from study inclusion 2. Death from any cause 3. Occurrence of ARDS	9.5% in the HCQ group had ECG changes requiring discontinuation of HCQ	No benefit of HCQ in severe COVID-19	2b
Lopinavir-ritonavir							
Cao [11]	Hubei, China	RCT (open-label) (*n* = 199)	1. Lopinavir-ritonavir (400 mg/100 mg) PO bid for 14 days (*n* = 99) 2. Standard care alone (*n* = 100)	Time to clinical improvement or discharge from hospital	Gastrointestinal events (anorexia, nausea, abdominal discomfort diarrhoea, acute gastritis, haemorrhage from lower digestive tract), self-limited skin eruptions	No benefit of lopinavir-ritonavir over standard care in clinical improvement or mortality in seriously ill COVID-19	1b
Zhou [12]	Wuhan, China	Retrospective cohort study (*n* = 191)	Lopinavir-ritonavir (dose unknown) (*n* = 41)	Nil	None reported	No improvement in duration of viral shedding	2b
Young [13]	Singapore	Case series (*n* = 18)	Lopinavir-ritonavir (400 mg/100 mg bid for up to 14 days)	Nil	Nausea, vomiting, diarrhoea, abnormal liver function test	Equivocal clinical benefit and duration of viral clearance	4
Kim [14]	Incheon, Seoul, Korea	Case report (*n* = 1)	Lopinavir-ritonavir (400 mg/100 mg, dose per day and duration unknown.	Nil	None reported	No conclusions can be drawn about efficacy or safety	5
Lim [15]	Goyang, Korea	Case report (*n* = 1)	Lopinavir-ritonavir (400 mg/100 mg bid; duration unknown	Nil	None reported	No conclusions can be drawn about efficacy or safety	5
Umifenovir (Arbidol®)							
Deng [16]	Guangdong, China	Retrospective cohort (*n* = 33)	1. Arbidol (0.2 g tid) and lopinavir-ritonavir (400 mg/100 mg bid) until RT-PCR negative for virus 3 times (*n* = 16) 2. Lopinavir-ritonavir only (*n* = 17)	RT-PCR negative for SARS-CoV-2 at days 7 and 14 from date of diagnosis, chest CT findings	Elevated bilirubin, mild gastrointestinal side effects	Arbidol with lopinavir-ritonavir might decrease the viral load of COVID-19 and delay progression of lung lesions	4
Wang [17]	Hubei, China	Retrospective cohort (*n* = 67)	Arbidol (0.4 g tid), median duration 9 days (*n* = 36)	Nil	None reported	Arbidol might improve rate of discharge from hospital and mortality rate	4
Remdesivir							
Grein [18]	USA, Japan, Italy, Austria, France, Germany, Netherlands, Spain, Canada	Prospective cohort study (*n* = 61)	Remdesivir (200 mg on day 1, then 100 mg od for 9 days)	Incidence of key clinical events, hospital discharge, adverse event, proportion of patients with clinical improvement.	Common: Elevated hepatic enzymes, diarrhoea, rash, renal impairment, hypotension. Serious adverse events: multiple organ dysfunction syndrome, septic shock, cute kidney injury, hypotension.	Clinical improvement observed in 68% of patients with severe COVID-19	2b
^a^COVID-19 Investigation Team [19]	Various states, USA	Case series (*n* = 12)	1. Remdesivir (200 mg once on day 1, then 100 mg od for 4–10 days until clinical improvement (*n* = 3) 2. No remdesivir (*n* = 9)	Nil	Transient gastrointestinal symptoms (nausea, vomiting, gastroparesis), elevated aminotransferase	No conclusions can be drawn about efficacy or safety	4
Lescure [20]	Paris, Bordeaux, France	Case series (*n* = 5)	Remdesivir (200 mg loading dose, then 100 mg od for 10 days) (*n* = 3)	Nil	Remdesivir discontinued in 1 patient due to combined elevated alanine aminotransferase and rash (uncertain drug adverse reaction)	No conclusions can be drawn about efficacy or safety	4
Holshue [21]	Washington, USA	Case report (*n* = 1)	Remdesivir (dose and duration unknown)	Nil	None reported	No conclusions can be drawn about efficacy or safety	5
Corticosteroids							
^a^Lu [22]	Hubei, Hangzhou, China	Meta-analysis	Systemic corticosteroids	1. Risk of mortality 2. Duration of pneumonia 3. Duration of hospitalisation 4. Duration of fever	None reported	Reduced duration of fever, but not mortality risk, duration of pneumonia. Associated with longer hospital stay.	2a
Zhou [23]	Hubei, China	Case series (*n* = 15)	Median hydrocortisone-equivalent dose of 400 mg per day after ICU admission, for average 9.5 days (*n* = 15)	Nil	None reported	No survival advantage in ICU patients with severe COVID-19, especially when complicated by ARDS and shock or multi-organ injury	4
Liu [24]	Hubei, China	Retrospective cohort study (*n* = 137)	IV methylprednisolone (30–80 mg/d for 3–5 days) (*n* = 40)	Nil	None reported	No observable benefit of corticosteroids	4
Heparin							
Tang [25]	Wuhan, China	Case-control study (*n* = 449)	1. LMWH (enoxaparin 40–60 mg/d, at least 7 days) (*n* = 94) 2. Unfractionated heparin (10,000–15,000 U/d, at least 7 days) (*n* = 5) 3. No heparin (*n* = 350)	Nil	None reported	Heparin may improve 28-day mortality in severe COVID-19 patients meeting sepsis-induced coagulopathy criteria or markedly elevated D-dimer	4
^a^Shi [26]	Wuhan, China	Retrospective cohort study (*n* = 42)	1. LMWH (*n* = 21) 2. Controls (*n* = 21)	Nil	None reported	Heparin can increase the proportion of lymphocytes and decrease IL-6 levels in severe COVID-19	4
Tocilizumab							
^a^Xu [27]	Anhui, China	Case series (*n* = 21)	Tocilizumab (400 mg, once dose) + LPV + methylprednisolone	Nil	None reported	Improved clinical status in severe to critically ill COVID-19	4
^a^Roumier [28]	Paris, France	Retrospective cohort (*n* = 30)	1. Tocilizumab (8 mg/kg, once, renewable once) (*n* = 30) 2. No tocilizumab	Nil	Hepatic cytolysis	Reduced ICU admission and requirement of mechanical ventilation in severe to critically ill COVID-19	4
Convalescent plasma							
Duan [29]	Wuhan, China	Prospective cohort (*n* = 10)	1 transfusion of 200 ml of convalescent plasma from donors with neutralising antibody titres > 1:640 (*n* = 10)	1. Safety of convalescent plasma transfusion 2. Improvement in clinical symptoms & laboratory parameters within 3 days of transfusion	None reported	Convalescent plasma was well-tolerated and could potentially improve clinical outcomes in severe COVID-19	4
Shen [30]	Shenzhen, China	Case series (*n* = 5)	2 consecutive transfusions of 200–250 ml of convalescent plasma with neutralizing antibody titre > 40	Nil	None reported	Improved clinical status in critically ill patients with ARDS	4
Ahn [31]	Seoul, Korea	Case series (*n* = 2)	2 transfusions of 250 ml of convalescent plasma at 12-h interval (optical density ratio for IgG: 0.532 & 0.586) (*n* = 2)	Nil	None reported	Favourable clinical outcome in critically ill patients with ARDS (combined with systemic corticosteroids)	5
Mesenchymal stem cell (MSC) treatment							
Leng [32]	Beijing, China	Pilot trial (*n* = 10)	1. MSC transplant (*n* = 7). 2. Placebo (*n* = 3)	1. Adverse events. 2. Cytokine variation, C-reactive protein, oxygen saturation. 3. Total lymphocyte count and subpopulations, chest CT, respiratory rate, patient symptoms	None reported	Symptoms, pulmonary function biochemistry apparently improved after MSC transplantation	4
^a^Liang [33]	Baoshan, China	Case report (*n* = 1).	MSC transplant 3 times, 3 days apart	Nil	None reported	No conclusion can be drawn	5

*LOE* Level of evidence, *tid* Three times a day, *od* Once a day, *RCT* Randomized controlled trial, *bid* twice a day, *ECG* Electrocardiogram, *SpO*_*2*_ Oxygen saturation, *ICU* Intensive care unit, *ARDS* Acute respiratory distress syndrome, *LMWH* Low molecular weight heparin, *RT-PCR* Reverse transcription polymerase chain reaction, *CT* Computed tomography; ^a^Published on pre-print medical server without peer review

The initial search identified a total of 1325 articles from PubMed and Embase. A search of the Cochrane Library database did not reveal any relevant articles. Studies in which combination drugs were used without distinguishing the primary drug studied were excluded. Studies reporting on traditional Chinese medicine were excluded due to the heterogenous nature of the drugs used and the active ingredient was not always known.

Thirty studies were eventually identified for the review after excluding duplicates and unsuitable studies. These studies reported clinical outcome with chloroquine or hydroxychloroquine (HCQ) (7 studies), lopinavir-ritonavir (5 studies), umifenovir (2 studies), remdesivir (4 studies), systemic corticosteroids (3 studies), low molecular weight heparin (LMWH) (2 studies), tocilizumab (2 studies), convalescent plasma (3 studies) and mesenchymal stem cell therapy (2 studies). We are aware of other potential investigational therapies such as interferon-alpha, ribavirin, intravenous immunoglobulin etc., but the literature search did not uncover any clinical studies investigating their individual use on COVID-19 patients and therefore these drugs are not included in this review.

## Clinical guidelines

Seven clinical guidelines on the management of COVID-19 by international or national professional bodies were identified. They are:

1. WHO: Interim guidance on clinical management of severe acute respiratory infection (SARI) when COVID-19 disease is suspected [3];
2. Infectious Diseases Society of America (IDSA): Guidelines on the treatment and management of patients with COVID-19 [35];
3. Surviving Sepsis Campaign: Guidelines on the management of critically ill adults with COVID-19 [36];
4. People’s Republic of China’s National Health Commission (NHC): Guidelines on the treatment of COVID-19 (7th edition) [37];
5. The Lombardy Section of the Italian Society of Infectious and Tropical Diseases (Società Italiana di Malattie Infettive e Tropicali) (SIMIT Lombardy Section): Vademecum for the treatment of people with COVID-19. Edition 2.0, 13 March 2020 [38];
6. The Netherlands’ Working Party on Antibiotic Policy (Stichting Werkgroep Antibiotica Beleid) (SWAB): Drug treatment options in patients with COVID-19 [39];
7. Belgium’s Sciensano (scientific institute of public health): Interim clinical guidance for adults with suspected or confirmed COVID-19 in Belgium [40].

The WHO, IDSA and Surviving Sepsis guidelines were generally in agreement of using investigational treatments only within the setting of clinical trials [3, 35, 36]. The IDSA recommended the use of chloroquine/HCQ with or without azithromycin, lopinavir-ritonavir, tocilizumab and convalescent plasma in the context of clinical trials due to current knowledge gaps [34]. The Surviving Sepsis guidelines specifically suggested against the routine use of lopinavir-ritonavir, convalescent plasma and intravenous immunoglobulins in critically ill COVID-19 patients (weak recommendation), and stated there was insufficient evidence to issue recommendations on the use of other anti-viral agents, recombinant interferons, chloroquine/HCQ or tocilizumab in critically ill COVID-19 patients [35]. However, guidelines from China, Italy, Netherlands and Belgium have listed some investigational drugs as potential adjuvant treatment options, whilst cautioning taking into consideration the individual risk of harm [37–40].

**Fig. 1:**
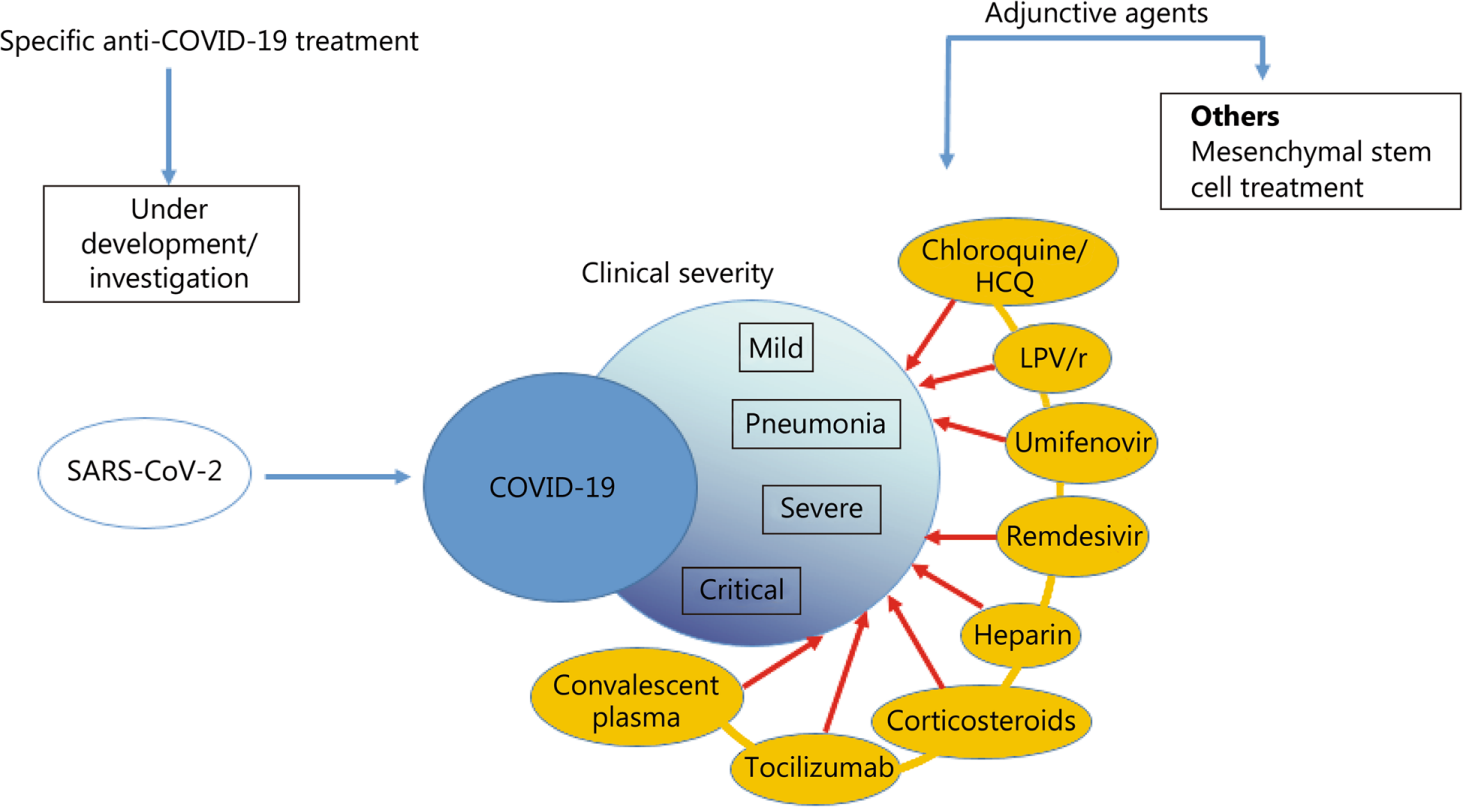
Summary of current adjunctive therapeutic agents used in clinical management of coronavirus disease (COVID-19). HCQ: Hydroxychloroquine; LPV/r: Lopinavir/ritonavir.

We have decided to organize these investigational treatments according to the clinical severity of COVID-19 where they may be utilized, based on the guidelines (Fig. 1). There is no general consensus on the clinical classification of COVID-19 and each guideline tends to use its own defined clinical categories of COVID-19. We therefore harmonized the categories across the various guidelines into “mild”, “pneumonia”, “severe” and “critical” groups according to case definitions put forth by the WHO (Table 2) [3]. This led to SWAB’s “moderately severe” group being re-categorized under the “severe” category to match WHO’s case definition. The guidelines from China, Italy, Netherlands and Belgium on the use of adjunctive treatments could then be compared based on fairly similar descriptions of clinical severity (Table 3).

**Table 2:** COVID-19 severity classifications across different guidelines harmonized according to WHO’s classification of severity, guided by WHO case definition

WHO classification and case definition	NHC (China)	SIMIT Lombardy section (Italy)	SWAB (Netherlands)	Sciensano (Belgium)
Mild	“Mild”	“Mild respiratory symptoms”	“Mild”	“Mild to moderate”
- Uncomplicated upper respiratory tract viral infection	- Mild clinical symptoms - No radiologic signs of pneumonia	- Fever (> 37.5 °C), cough, no dyspnoea	- No supplemental oxygen required	- no oxygen requirement or no evidence of pneumonia
Pneumonia	“Common” or “Regular”	“Moderate respiratory symptoms”	Nil	
- Pneumonia but no signs of severe pneumonia - No need for supplemental oxygen	- Fever, symptoms of respiratory tract infection - Signs of pneumonia on imaging	- Fever (> 37.5 °C), cough, mild to moderate dyspnoea, and/or - Pneumonia on chest x-ray - Mild respiratory symptoms in age >70 years and/or co-morbidities with increased mortality risk		
Severe	“Severe”	Nil	“Moderately severe”	“Severe”
- Fever or suspected respiratory infection, plus 1 of the following: • RR > 30/min; • Severe respiratory distress • SpO_2_ ≤ 93% on room air	- Dyspnoea, respiratory rate > 30/min - SpO_2_ < 93% at rest, or - PaO_2_/FiO_2_ ratio < 300 mmHg		- Requires monitoring in ward - Supplemental oxygen required	≥1 of the following: - RR ≥30/min - SpO_2_ ≤ 93% - PaO_2_/FiO_2_ ratio < 300 mmHg - Lung infiltrates > 50% of the lung field within 24–48 h
Critical	“Critical”	“Critically ill”	“Very severe”	“Critical”
- ARDS, or - Sepsis with acute organ dysfunction	- Respiratory failure requiring mechanical ventilation, or - Presence of shock, or - Multi-organ failure requiring monitoring in ICU	- ARDS - Respiratory failure - Haemodynamic failure (re-classified under WHO’s “critical” category)	- Monitoring in ICU required, or - ECMO required, or - Clinical deterioration from moderate severity with initial anti-viral therapy	≥1 of the following: - ARDS - Sepsis - Altered consciousness - Multi-organ failure

*WHO* World Health Organization, *NHC* National Health Commission, *SIMIT* Società Italiana di Malattie Infettive e Tropicali (Italian Society of Infectious and Tropical Diseases), *SWAB* Stichting Werkgroep Antibiotica Beleid (Working Party on Antibiotic Policy), *RR* Respiratory rate, *SpO*_*2*_ Peripheral oxygen saturation, *PaO*_*2*_ Partial pressure of arterial oxygen, *FiO*_*2*_ Fraction of inspired oxygen, *ARDS* Acute respiratory distress syndrome, *ICU* Intensive care unit, *ECMO* Extra-corporeal membrane oxygenation

**Table 3:** Summary of national guidelines in the use of investigational adjunctive treatments in COVID-19

Severity of COVID-19 (WHO classification)	NHC (China)	SIMIT Lombardy section (Italy)	SWAB (Netherlands)	Sciensano (Belgium)
Mild	Symptomatic treatment Other general treatments: • Interferon-alpha (5 million units or equivalent dose added to 2 ml sterile water, delivered via nebulizer bid) • Lopinavir-ritonavir (400 mg/100 mg bid; not > 10 days) • Ribavirin (500 mg bid/tid, not > 10 days)(recommended in combination with interferon or lopinavir-ritonavir) • Chloroquine phosphate (500 mg bid for 7 days in adults 18–65 years and body weight > 50 kg; 500 mg bid for days 1–2, followed by 500 mg od for days 3–7 in adults < 50 kg) • Umifenovir (200 mg tid, not > 10 days)	Symptomatic treatment In age > 70 years old and/or co-morbidities • Consider lopinavir-ritonavir (400 mg/ 100 mg bid) + Chloroquine (500 mg bid) or HCQ (200 mg bid) for 5–20 days) Alternatives to lopinavir-ritonavir: • Darunavir + ritonavir (800 mg/ 100 mg od), or • Darunavir + cobicistat (800 mg/ 150 mg od)	Symptomatic treatment	Symptomatic treatment • Consider starting HCQ (400 mg at diagnosis, then 400 mg 12 h later, followed by 200 mg bid up to day 5) or • chloroquine base (10 mg/kg at diagnosis, 5 mg/kg 12 h later, followed by 5 mg/kg bid up to day 5) or • chloroquine phosphate (1000 mg at diagnosis, then 500 mg bid, followed by 300 mg bid up to day 5) (including age > 65 years and/or underlying end-organ dysfunction)
Pneumonia		• Lopinavir-ritonavir (400 mg/100 mg bid) + Chloroquine (500 mg bid) or HCQ (200 mg bid) for 5–20 days) BCRSS^*^ score ≥ 2, consider adding: - Dexamethasone 20 mg/day for 5 days, then 10 mg/d for 10 days (discuss with intensivist) and/or - Tocolizumab	Nil	
Severe	• Convalescent plasma • Tocolizumab (extensive lung disease, increased IL-6; prohibited in active tuberculosis)(IV, 4–8 mg/kg, maximum 2 cumulative doses) • Glucocorticoids (not exceeding equivalent of methylprednisolone 1–2 mg/(kg·d), for 3–5 days) • Xuebijing (TCM)(100 ml bid) • Probiotics	Nil mentioned	• Chloroquine (600 mg loading dose, 300 mg 12 h later, followed by 300 mg bid on days 2–5 or • HCQ (400 mg bid loading dose, then 200 mg bid on days 2–5) Consider switching or adding remdesivir if insufficient response or clinical deterioration	• Prophylactic LMWH • Start HCQ or chloroquine (above dose) • Consider lopinavir-ritonavir (400 mg/100 mg bd for 14 days) only if HCQ/ chloroquine is contraindicated and if it can be administrated with 12 days of symptom onset
Critical		• Remdesivir (IV 200 mg loading dose on day 1, maintenance dose 200 mg/d from day 2–10) + chloroquine/HCQ (above dose) or • Lopinavir-ritonavir + chloroquine/HCQ (above dose) ARDS: • Dexamethasone 20 mg/d for 5 days, then 10 mg/d for 5 days; to initiate within 24 h of ARDS diagnosis (discuss with intensivist) and/or • Tocilizumab	• Chloroquine/ HCQ + remdesivir (200 mg loading dose on day 1, then 100 mg daily for days 2–9) or • Remdesivir alone	• Remdesivir (200 mg loading dose within 30 min, followed by 100 mg daily for 2–10 days) • Consider HCQ/ chloroquine if remdesivir unavailable • IL-6 inhibitors should only be used in clinical trials

BCRSS^*^ Brescia-COVID Respiratory Severity Scale, based on 4 criteria: patient wheezing or unable to speak in full sentences while at rest/with minimal effort; respiratory rate > 22, PaO_2_ < 65 mmHg or SpO_2_ < 90%; worsening repeat chest X-ray (not externally validated), *WHO* World Health Organization, *NHC* National Health Commission, *SIMIT* Società Italiana di Malattie Infettive e Tropicali, *SWAB* Stichting Werkgroep Antibiotica Beleid, *HCQ* Hydroxychloroquine, *bid* Twice a day, *tid* Three times a day, *RR* Respiratory rate, *SpO*_*2*_ Peripheral oxygen saturation, *PaO*_*2*_ Partial pressure of arterial oxygen, *FiO*_*2*_ Fraction of inspired oxygen, *TCM* Traditional Chinese medicine, *IV* Intravenous, *LMWH* Low molecular weight heparin, *ARDS* Acute respiratory distress syndrome, *DVT* Deep venous thrombosis, *ECMO* Extracorporeal membrane oxygenation

## Mild illness and pneumonia

All guidelines (except for the Surviving Sepsis guidelines which were specifically for critically ill COVID-19 patients) unanimously recommended symptomatic treatment for mild cases, which were generally defined as uncomplicated respiratory tract infections and may not require hospitalization [3, 35, 37–40]. As adjuncts to this, the NHC, SIMIT Lombardy section and Sciensano guidelines recommended considering the use of chloroquine/HCQ and/or lopinavir-ritonavir, including for those in the pneumonia category [37, 38, 40]. The United States Food and Drug Administration (US FDA) has authorized the emergency use of chloroquine and HCQ from the Strategic National Stockpile for treatment of hospitalized adults and adolescents with COVID-19 for whom a clinical trial is not available or participation is not feasible [41]. The NHC guidelines also recommended umifenovir in this category, albeit being the only guideline to suggest the use of this drug. Therefore, chloroquine/HCQ, lopinavir-ritonavir and umifenovir will be discussed in this section.

### Chloroquine/Hydroxychloroquine

Chloroquine is used in both treatment and chemoprophylaxis against malaria. HCQ, an analogue of chloroquine, is used in autoimmune conditions such as systemic lupus erythematosus and rheumatoid arthritis. Both drugs have shown in-vitro activity against SARS-CoV-2, with HCQ possibly being the more potent of the two [42, 43]. Their anti-viral mechanisms of action are not clear, but have been postulated to include inhibition of the pH-dependent steps of viral replication and immunomodulation via inhibition of tumor necrosis factor-alpha and interleukin-6 (IL-6) [44]. Given that both drugs have been around for decades, they are generally affordable and their safety profiles are well-established, they are attractive candidates as potential anti-COVID-19 treatments.

The study that arguably sparked much global interest in HCQ as a potential treatment for COVID-19 was Gautret et al.’s non-randomized case-control study in France, which compared HCQ (*n* = 14) and HCQ plus azithromycin for prevention of bacterial superinfection (*n* = 6) against a control group (*n* = 16) [4]. The inclusion criteria was age > 12 years and confirmed SARS-CoV-2 carriage in nasopharyngeal sample at admission no matter their clinical status. The main outcome of the trial was virus carriage in nasopharyngeal swabs at day 6. At day 6 of treatment, all patients in the HCQ plus azithromycin group tested virus-free, compared to 57.1% in the HCQ-alone group and 12.5% of the control group (*P* < 0.001). However, 1 patient who tested negative on day 6 subsequently tested positive on day 8. Clinical benefit was also not assessed. Six patients who dropped out from the HCQ group were not included in the analysis, including patients who could not tolerate the drug, were escalated to intensive care unit (ICU) or eventually died. No adverse effects were documented.

A couple of reports have noted favorable outcomes with chloroquine/HCQ. In a randomized controlled trial (RCT) comparing HCQ (*n* = 31) versus no HCQ (*n* = 31) in mild to common COVID-19 (NHC criteria) patients, Chen et al. [5] reported that the HCQ group had shorter time to clinical recovery and radiologic improvement in pneumonia. However, the sample size was small, the follow-up period was short (5 days following enrolment in the study) and the statistical analysis of the results was not clear. Another report stated that results from more than 100 patients from clinical trials investigating chloroquine use in COVID-19 patients had shown benefits in clinical improvement and virologic clearance compared to controls [45]. However, details of these patients were not reported in this paper.

Studies with less encouraging results have also emerged. Chen et al. [6] reported their preliminary results from a small non-blinded RCT in China comparing HCQ (*n* = 15) against a control group (*n* = 15) in patients with common COVID-19 (NHC criteria). Following 7 days of treatment, throat swabs were negative for the virus in 86.7% of the HCQ group compared to 93.3% of the control group (*P* > 0.05). The mean duration from hospitalization to viral clearance was comparable in both groups. There were 4 adverse events in the treatment group: 2 cases of diarrhea, 1 case of disease progression and 1 case of transiently elevated aspartate aminotransferase. The authors noted that the overall prognosis of common COVID-19 appeared to be good, and HCQ treatment in common COVID-19 patients did not appear to have clear benefits.

Magagnoli et al. [7] performed a retrospective review of their cohort of 368 males from the US Veterans Health Administration medical centers who received either HCQ, HCQ plus azithromycin or no HCQ for COVID-19 of varying severities. They observed that the risk of death from any cause was higher in the HCQ group (adjusted hazard ratio (*HR*) = 2.61; 95% CI: 1.10–6.17; *P* = 0.03) compared to the no-HCQ group. The risk of ventilation was no different in patients who did not receive HCQ compared to those who received HCQ alone (adjusted *HR*, 1.43; 95% CI: 0.53–3.79; *P* = 0.48) or with azithromycin (adjusted *HR =* 0.43; 95% CI: 0.16–1.12; *P* = 0.09). The risk of death after ventilation also was not significantly different across the 3 groups. However, limitations included the retrospective nature of the study, the select patient population of male, predominantly African American veterans, differing baseline demographics across all 3 groups and inclusion of a spectrum of COVID-19 severity.

The SIMIT Lombardy section, SWAB and Sciensano guidelines included chloroquine/ HCQ in treatment of severe to critically ill COVID-19 patients, each with varying dosing regimens [38–40]. A concern with chloroquine is its narrow therapeutic window and consequent risk of toxicity. Chloroquine and HCQ have strong tissue tropism for the kidney and liver. At higher cumulative doses, such as with ICU patients who are far more likely to have renal and/or hepatic dysfunction, the risk of cardiotoxicity, prolonged QT interval and arrhythmia is substantially increased [46]. Long term exposure to chloroquine/HCQ carries added risks of retinopathy, maculopathy and cardiomyopathy, therefore short courses of chloroquine are generally recommended by professional bodies when used in COVID-19. The expert consensus on chloroquine by the multicenter collaboration group of the Department of Science and Technology and Health Commission of Guangdong Province recommended monitoring with daily blood counts, electrolytes and cardiac enzymes every other day; electrocardiogram pre-treatment, and 5 and 10 days after starting treatment [47]. Although the American Academy of Ophthalmology does not recommend retinal screening before short-term use of chloroquine [48], patients should be asked about visual changes during treatment. The use of chloroquine with concurrent macrolides (including azithromycin) and quinolone was not recommended in view of risks of prolonged QT interval [47].

Four studies have investigated the use of chloroquine/HCQ in severe COVID-19 patients. Borba et al. [8] released their preliminary findings of high dose (600 mg twice a day for 10 days) versus low dose (450 mg twice a day on day 1, followed by 450 mg daily on days 2–5) chloroquine diphosphate in the treatment of severe COVID-19 in a randomized, double-blinded, phase IIb clinical trial. The study halted recruitment of patients into the high dose chloroquine arm after just 6 days into the study when authors noted that more patients in this arm demonstrated prolonged QTc and a trend towards more deaths. Two patients died from ventricular tachycardia. Molina et al. [9] noted in their prospective case series of 11 severe COVID-19 patients that HCQ plus azithromycin, in doses similar to that used in the study by Gautret et al. [4], saw 1 patient discontinue treatment due to prolonged QT interval. Moreover, viral clearance and clinical outcome was not improved by this drug combination. Mahévas et al. [10] observed in their cohort of 181 patients with severe COVID-19 that HCQ did not significantly reduce ICU admission, death at day 7 after hospitalization or reduce the incidence of ARDS compared to those who did not receive HCQ. Furthermore, 8 (9.5%) of 84 patients in the HCQ group discontinued HCQ after a media of 4 days due to prolonged QT interval or first-degree atrioventicular block. Perinel et al. [49] attempted to address the issue of HCQ dosing in severe to critically ill COVID-19 patients by studying the pharmacokinetic properties of HCQ in 13 COVID-19 ICU patients. The study population had a median renal function of 79.6 ml/min. Twelve patients were mechanically ventilated, 4 had moderate or severe renal failure, 1 required renal replacement therapy and 1 required extracorporeal membrane oxygenation (ECMO). On a regimen of oral HCQ 200 mg three times daily, only 8 (61%) patients achieved the minimum therapeutic level of 1 mg/L and 2 (15.4%) patients exceeded the maximum therapeutic level of 2 mg/L. HCQ was withdrawn in 2 (15.4%) patients due to prolonged QT interval. The HCQ blood levels of the patient on ECMO increased more slowly compared to other patients. The authors recommended 800 mg once on the first day to rapidly achieve therapeutic levels, followed by 200 mg twice daily for 7 days in ICU patients.

Overall, the findings from these studies suggest limited benefit from chloroquine/HCQ in COVID-19 in general. The discrepancies in findings may stem from different patient populations, differences in inclusion criteria, paucity of long-term follow-up data, differences in drug dosages, lack of control group and inclusion of azithromycin in the some studies. These preliminary findings will need to be confirmed with large scale RCTs when they are completed. More clinical studies are also needed to establish the safety and dosing of chloroquine/ HCQ, especially if to be used in severe to critically ill patients.

### Lopinavir-ritonavir

Lopinavir is a human immunodeficiency virus (HIV) type-1 aspartate protease inhibitor. Ritonavir inhibits CYP3A-mediated metabolism of lopinavir, thereby increasing the serum concentration of the latter and is therefore often given in combination. Lopinavir-ritonavir has been used off-label during SARS and MERS outbreaks. A systematic review of lopinavir-ritonavir use in SARS and MERS coronaviruses reported two retrospective matched cohort studies showing that lopinavir-ritonavir improved clinical outcome when given early in SARS patients, and lopinavir-ritonavir alone or given in combination with interferon improved clinical outcome of some MERS patients [50].

A literature search revealed 5 in-vivo studies of lopinavir-ritonavir use in COVID-19 patients. The only RCT was an open-label study in China by Cao et al. [11], comparing standard treatment (*n* = 100) versus standard treatment together with lopinavir-ritonavir (*n* = 99). The study population was patients with arterial oxygen saturation (SaO_2_) ≤94% on room air or a ratio of partial pressure of arterial oxygen (PaO_2_) to fraction of inspired oxygen (FiO_2_) < 300 mmHg, which was close to NHC’s definition of severe COVID-19. The authors did not observe significantly improved clinical outcomes in the lopinavir-ritonavir group. The time to clinical improvement, mortality at 28 days and viral RNA load or detectability of viral RNA at various time points was not significantly different between the 2 groups. The lopinavir-ritonavir group reported 4 serious adverse events (2 acute gastritis, 2 haemorrhage of the lower digestive tract); 13 patients were unable to complete the full course due to anorexia, nausea, abdominal discomfort, or diarrhea. Two patients had self-limited skin eruptions. The high overall mortality rate (22.1%) in this trial was noted to be a potential confounder, as patients may have been too ill to respond to the drug.

A retrospective cohort study of 191 COVID-19 patients in China by Zhou et al. [12] observed that of the 29 patients who received lopinavir-ritonavir at a median time of 14 days from the onset of illness and were eventually discharged, the duration of viral shedding was not significantly shortened (median duration: 22.2 days). As a comparison, patients with severe disease tended to shed the virus for a median duration of 19.0 days and those with critical disease was a median of 24.0 days.

In a descriptive case series of 18 COVID-19 patients in Singapore, 5 patients who required supplemental oxygen were administrated lopinavir-ritonavir within 1 to 3 days of desaturation [13]. Three patients had reduction in oxygen requirements within 3 days of treatment, and 2 tested negative for the virus within 2 days of treatment. Two patients progressively worsened, of whom 1 required invasive mechanical ventilation. Four patients developed nausea, vomiting and/or diarrhoea; 3 had abnormal liver function tests and only 1 patient managed to complete the 14-day course of treatment. The authors noted that lopinavir-ritonavir had equivocal clinical benefits and duration of viral clearance.

There were 2 case reports from Korea. Kim et al. [14] reported a young healthy female with COVID-19 requiring oxygen supplementation up to 6 L/min who received lopinavir-ritonavir on day 4 of illness. Her fever improved from day 10 of illness; dyspnoea, oxygen requirement and radiologic findings improved from day 14. The viral load was not measured. Lim et al. [15] reported a middle-aged healthy male who was initiated on lopinavir-ritonavir on day 10 of illness. The degree of respiratory support required was unknown. The patient’s viral load decreased the day after administration of lopinavir-ritonavir until little or no virus titers were detected, although the authors conceded this could be due to the natural course of the disease rather than the effect of the drug. No adverse effects from lopinavir-ritonavir were reported in both case reports.

From the limited evidence, there appears to be equivocal benefit of lopinavir-ritonavir on clinical improvement and viral clearance. Apart from the adverse effects encountered in these clinical studies, lopinavir-ritonavir is additionally known to cause liver injury, pancreatitis, leukopaenia, anaemia, severe cutaneous eruptions, QT prolongation and the potential for drug interactions from inhibition of CYP3A enzymes [51, 52].

### Umifenovir

Umifenovir is small indole-derivative molecule that has broad-spectrum antiviral properties, including Influenza A and B [53]. It blocks viral fusion with the target membrane, thus providing viral entry into target cells. It is approved for prophylaxis and treatment of influenza in Russia and China.

Deng et al. performed a retrospective non-randomized cohort study of 33 patients in China, stratified into 16 patients who received oral umifenovir and lopinavir-ritonavir versus 17 patients who received lopinavir-ritonavir without umifenovir [16]. At day 7 of treatment, 75% in the combination group tested negative for the virus, compared with 35% in the lopinavir-ritonavir-only group (*P* < 0·05). Chest CT findings improved for 69% in the combination group compared with 29% patients in the lopinavir-ritonavir-only group (*P* < 0·05). At day 14, 94% in the combination group tested negative compared to 53% in the lopinavir-ritonavir-only group (*P* < 0·05). None of the patients developed acute respiratory failure, required invasive ventilation or vasopressor therapy during the treatment. Adverse effects recorded included hyperbilirubinemia (68.7%) and mild gastrointestinal symptoms (43.7%) such as diarrhoea and nausea. The authors concluded that combination therapy might decrease the viral load of COVID-19 and delay progression of lung lesions. Potential confounders included the use of other drugs in patients in both groups (immunoglobulin therapy, corticosteroids, non-specified anti-virals).

Wang et al. performed a retrospective cohort study of 67 COVID-19 patients in China, who were stratified according to the lowest recorded peripheral oxygen saturation (SpO_2_) into the SpO_2_ ≥ 90% group (*n* = 55) and the SpO_2_ < 90% group (*n* = 12) [17]. Thirty-two (58.2%) patients in the SpO_2_ ≥ 90% group received umifenovir while 4 (33.3%) patients in the SpO_2_ < 90% received umifenovir. The authors observed that 12 (33%) of 36 patients in the umifenovir-treated group had been discharged, compared to 6 (19%) of 31 patients in the no-umifenovir group who had been discharged (*P* = 0.03). The mortality rate of this cohort was 7.5%; all who had received umifenovir survived. No adverse events were reported. The authors commented that umifenovir could improve the rate of discharge from hospital and mortality rate of COVID-19 patients. However, 88.9% of the patients who received umifenovir belonged to the SpO_2_ ≥ 90% group, which had a mean age of 37.0 years and could arguably have better prognosis than the SpO_2_ < 90% group, which had a mean age of 70.5 years.

From the 2 cohort studies, no clear conclusion could be drawn about the benefit of administering umifenovir. Little is also known about the adverse effects of umifenovir.

## Severe and critical illness

The SIMIT Lombardy section, SWAB and Sciensano guidelines had recommended the consideration of remdesivir as compassionate use in critically ill patients [38–40]. The WHO and Sciensano had recommended the use of heparin as prophylaxis against venous thromboembolism in this group of patients [3, 40]. The use of systemic corticosteroids had been mentioned under this category by several guidelines and appeared to be controversial. The WHO recommended against routine corticosteroids for pneumonia outside clinical trials, but did not comment on their role in ARDS [3]. In patients with ARDS, the SIMIT Lombardy section and Surviving Sepsis guidelines suggested considering of a short course of systemic corticosteroids [36–38], whereas the IDSA recommended their use only in the context of a clinical trial [35]. Tocolizumab had been mentioned as an option in the NHC and SIMIT Lombardy section guidelines [37, 38], but recommended only in the context of clinical trials by the Sciensano guidelines [40]. The NHC guidelines had also suggested considering convalescent plasma in this category [37]. These adjunctive treatments will be discussed in this section.

### Remdesivir

Remdesivir is a novel nucleotide analogue prodrug which is incorporated into nascent viral RNA chains, causing premature termination of RNA transcription [54]. It was developed for use against the Ebola virus, an epidemic RNA virus [55]. However, its use was suspended after a RCT evaluating the safety and efficacy of 3 monoclonal antibodies and remdesivir) terminated random assignment to remdesivir due to a clear reduction in survival in this treatment group [55]. In-vitro studies had shown that remdesivir effectively inhibited the replication of SARS-CoV and MERS-CoV [56, 57], and appeared to have effect on SARS-CoV-2 replication as well in non-human cells [42]. Remdesivir is not approved to treat any condition by regulatory agencies, including the US FDA or the European Medicines Agency.

Grein et al. [18] reported the outcome of an open-label cohort study of 61 COVID-19 patients from 9 countries who received remdesivir on a compassionate-use basis. The inclusion criteria were hospitalized COVID-19 patients with SpO_2_ ≤ 94% on ambient air or supplemental oxygen, creatinine clearance > 30 ml/min, serum aminotransferases less than 5 times the upper limit of normal and not on other investigational drugs for COVID-19. Eight patients were excluded due to missing or erroneous data. The study population included 34 (64%) patients on invasive ventilation and 4 (8%) patients on ECMO. Over 18 days, 36 of 53 patients (68%) showed an improvement in the category of oxygen support, including 17 (57%) of 30 patients who were extubated. Eight of 53 patients (15%) showed worsening. The mortality rate of the cohort was 13%, including 6 of 34 patients on invasive ventilation and 1 of 19 patients on non-invasive oxygen support. Adverse events were reported in 60% of the study population and were generally more common in patients on invasive ventilation. The most common adverse events were increased hepatic enzymes, diarrhea, rash, renal impairment and hypotension. Serious adverse events included multiple- organ-dysfunction syndrome, septic shock, acute kidney injury, and hypotension. Four (8%) patients discontinued remdesivir treatment due to worsening pre-existing renal failure, multiple organ failure, elevated aminotransferases and maculopapular rash. Some limitations of the study were the lack of pre-defined sample size, short duration of follow-up, lack of data on viral load to determine anti-viral effects and lack of control group. Notably, the pharmaceutical company that developed remdesivir was responsible for the funding, study design, approving the patient selection and drafting the manuscript.

The COVID-19 Investigation Team in the United States described 12 COVID-19 patients, of whom 3 (who appeared to fulfil the WHO severe illness criteria) received remdesivir for 4 to 10 days at the time of clinical worsening [19]. The efficacy of remdesivir in clinical improvement or viral clearance was not known as these were not outcome measures. Following initiation of remdesivir, all 3 patients experienced elevated aminotransferases and transient gastrointestinal symptoms, including nausea, vomiting, gastroparesis or rectal bleeding, although the patient with rectal bleeding was later stool-tested positive for Giardia and Clostridiodes difficile. However, it was also described that the 3 patients tolerated treatment with remdesivir. This study was published on a preprint server without peer review.

Lescure et al. [20] reported their case series of 5 COVID-19 cases in France, of whom 3 with at least severe illness requiring ICU monitoring received remdesivir. In 2 patients, remdesivir was administered at day 11–15 of illness. One patient discontinued remdesivir after 4 days due to raised alanine aminotransferase 3 times higher the higher limit of normal and maculopapular rash. Both patients recovered and were discharged. The third patient who was administered remdesivir was elderly and critically ill with multiorgan failure and eventually died.

Holshue et al. [21] reported the first case of COVID-19 in the United States. A young healthy male received remdesivir on day 11 of illness when he continued to demonstrate ongoing fever and atypical pneumonia on chest X-ray. The following day, his clinical condition improved and supplemental oxygen was discontinued. He remained hospitalized at time of conclusion of data collection. Side effects from remdesivir were not reported.

Based on the current evidence, no collective conclusion can be drawn about the therapeutic efficacy or safety profile of remdesivir in the treatment of COVID-19. Elevated liver enzymes were a common feature reported in 3 of the 4 studies.

### Systemic corticosteroids (against routine use)

A search of the literature uncovered 3 articles examining the role of corticosteroids in patients with COVID-19. In a meta-analysis of systemic corticosteroid use in COVID-19 patients by Lu et al. [22], the pooled results from 5 cohort studies found that corticosteroids did not reduce the risk of mortality (relative risk (*RR*) = 2.0, 95% CI: 0.7–5.8, *I*^2^ = 90.9%), shorten the duration of pneumonia (weighted mean difference (WMD) = − 1.0 day, 95% CI: − 2.9 - 0.9), or shorten hospital stay (WMD = 2.4 days, 95% CI: 1.4–3.4, *I*^2^ = 0.0%) in COVID-19 patients. However, the duration of fever was significantly lower in COVID-19 patients who received corticosteroids than patients who did not receive corticosteroids (WMD = − 3.2 days, 95% CI: − 3.6 to − 2.9). The authors concluded that the evidence did not support routine use of systemic corticosteroids in COVID-19.

Two other studies were identified that were not included in the above meta-analysis. Zhou et al. [23] described the efficacy of corticosteroids in a cohort of 15 critical COVID-19 patients (NHC criteria) with moderate to severe ARDS. All had received anti-virals and/or antibiotics without improvement. Corticosteroids (median hydrocortisone-equivalent dose of 400 mg/d) were initiated upon ICU admission for an average of 9.5 days. The authors observed that while corticosteroids improved arterial oxygenation (SaO_2_) and PaO_2_/FiO_2_ ratio in the first 3 to 5 days which could theoretically be further augmented with invasive ventilation, overall survival was not improved. The mortality rate of the study was 46.7%, compared with the 57.6% mortality rate of MERS ICU patients who did not receive corticosteroids. Corticosteroids did not exert a survival advantage in 7 patients with concomitant ARDS, shock or multi-organ failure, who all eventually expired.

Liu et al. [24] described their cohort of 137 COVID-19 patients, of whom 24.8% required non-invasive ventilation. None of the patients required invasive ventilation or ICU management. The mortality rate of this cohort was 11.7%. Forty (29.2%) patients who had persistently high fever or significant short-term disease progression on chest imaging were administered intravenous methylprednisolone (30–80 mg/d, for 3 to 5 days), with a view to inhibit cytokine storm and promote resorption of exudates. The authors observed that low dose, short course of intravenous methylprednisolone (30–80 mg/d, for 3 to 5 days) did not appear to improve patient outcomes. Potential confounders included the use of other drugs such as anti-virals (not specified) and gamma-immunoglobulin.

From the three studies, no clear conclusion could be drawn on giving corticosteroids in severe to critically ill COVID-19 patients. In general, the concern with the use of systemic corticosteroids in this group is with increased likelihood of harm and lack of clear benefit based on evidence from corticosteroid treatment in SARS, MERS and other severe respiratory virus infection. A systemic review of SARS treatment reported 29 studies on corticosteroid use, of which 25 were inconclusive and 4 demonstrated possible harm [58]. A multi-center retrospective cohort study of 309 ICU patients with MERS noted that patients who received corticosteroids were associated with delayed viral clearance and lack of survival benefit [59]. A meta-analysis of patients with seasonal and pandemic influenza from 3 Asian cohorts observed that corticosteroid therapy was associated with superinfection and increased mortality [60]. Based on this, the overall evidence generally favours against routine use of corticosteroids in critically ill patients, and the decision to administer corticosteroids should be made on an individual basis following discussion with the intensivist.

### Low molecular weight heparin

Severe to critically-ill patients can be complicated by sepsis-induced coagulopathy, disseminated intravascular coagulation or venous thromboembolism from prolonged bedrest. However, critically-ill COVID-19 patients appear to be particularly predisposed towards thrombotic complications. A Dutch study of 184 critically-ill COVID-19 patients in ICU noted a 31% incidence of thrombotic complications including ischaemic stroke, systemic arterial embolism and myocardial infarction [61]. Similarly, a study of 81 critically-ill COVID-19 patients in ICU in China observed a 25% incidence of venous thromboembolic events [62]. Indicators of pro-coagulation state such as elevated D-dimer, fibrin degradation product levels, inflammatory markers, and prolonged prothrombin time and activated partial thromboplastin time in this population are associated with increased risk of mortality [12, 63, 64].

Tang et al. [25] evaluated their cohort of 449 patients with severe COVID-19, of whom 99 patients received mainly LMWH for at least 7 days. There was no difference in the 28-day mortality rate between heparin users and non-users (30.3% vs 29.7%, *P* = 0.910). However, in patients meeting sepsis-induced coagulopathy criteria or having markedly increased D-dimer, the 28-day mortality of heparin recipients was significantly lower than that of non-recipients (40.0% vs 64.2%, *P* = 0.029).

Shi et al. [26] performed a retrospective cohort study of 42 patients with severe COVID-19, with 21 patients in the LMWH group and no-heparin group each. The authors found that LMWH had no effect on the duration of viral clearance and duration of hospitalization. Biochemically, the LMWH group had higher proportion of lymphocytes and reduced IL-6 compared to the control group. This study was published in a preprint medical server without peer review.

The WHO and Sciensano guidelines recommended the use of prophylactic LMWH or heparin against venous thromboembolism in severe to critically ill COVID-19 patients [3, 40]. In addition, the International Society on Thrombosis and Haemostasis recommended that all hospitalized COVID-19 patients, not just those in ICU, should receive prophylactic LMWH in the absence of contraindications (active bleeding, platelet count < 25 × 10^9^/L) [65].

### Interleukin-6 inhibitors (Tocilizumab)

Tocilizumab is a humanized immunoglobulin that blocks the IL-6 receptor. It is licensed in the US and Europe for chimeric antigen receptor T-cell-induced severe or life-threatening cytokine release syndrome. It is hypothesized to be effective in suppressing the cytokine storm syndrome associated with severe or critical COVID-19 [66].

There are 2 studies on tocilizumab use in COVID-19, Xu et al. [27] reported a case series of 21 patients from China with severe or critical COVID-19 who received tocilizumab in addition to LPV and methylprednisolone. The authors observed resolution of fever the following day and subsequent improvement in clinical symptoms and oxygen saturation. Inflammatory markers and chest CT also showed improvement within a week in majority of patients. Roumier et al. [28], in their study of 30 severe to critically ill patients in France who received tocilizumab, noted that tocilizumab significantly reduced the requirement of mechanical ventilation compared to controls (weighted odds ratio (*OR*) = 0.42; 95% CI: 0.20–0.89; *P* = 0.025) and reduced the risk of ICU admission in those treated outside ICU (weighted *OR* = 0.17; 95% CI: 0.06–0.48; *P* = 0.001). However, there was no statistically significant difference in reduction of mortality after weighted analysis (*OR* = 0.25; 95% CI: 0.05–0.95; *P* = 0.04). Both studies were published in a preprint server without peer review.

From a systematic review and meta-analysis of RCTs conducted in patients with rheumatoid arthritis, tocilizumab is associated with an increased risk of infectious respiratory adverse events [67]. It carries a FDA black box warning of serious infections including tuberculosis, bacterial, invasive fungal and viral infections.

Only 2 guidelines included tocilizumab in the management algorithm. The NHC guidelines recommended the use of tocilizumab in severe COVID-19 with extensive bilateral lung disease and elevated IL-6 [37]. The SIMIT Lombardy section guidelines suggested tocilizumab in critically ill patients with ARDS [38]. However the current evidence is insufficient to support the use of tocilizumab outside clinical trials.

### Convalescent plasma

Convalescent plasma is blood plasma from a person who has recovered from an infection and contains neutralizing antibodies against the offending agent. It is considered a form of passive immunotherapy. Convalescent plasma has been explored as a treatment option in SARS and severe influenza; a meta-analysis noted it may reduce mortality, although many studies were of low quality and lacked control groups [68]. Currently, the only guideline that includes the use of convalescent plasma in its algorithm is from the NHC [37].

A literature search found 3 articles examining the use of convalescent plasma in COVID-19 patients. Duan et al. [29] reported on 10 patients with severe COVID-19 (NHC criteria) in China who received one dose of 200 ml convalescent plasma from recovered donors with neutralizing antibody titres above 1:640. Patients additionally received various treatments including umifenovir, remdesivir, ribavirin, peramivir and methylprednisolone. Clinical symptoms improved within 3 days, and general improvements in chest CT appearance and lymphocyte counts were noted. The viral load became undetectable within 6 days of transfusion in 7 patients with pre-transfusion viraemia. Compared to 10 controls, the treatment group had greater proportions of patients discharged, improved and no deaths. No adverse events were reported.

Shen et al. reported a case series of 5 critically ill (NHC criteria) COVID-19 patients with ARDS who received convalescent plasma containing neutralizing antibodies in China [30]. Each patient received 2 consecutive transfusions of 200 to 250 ml of ABO-compatible convalescent plasma each time, on the same day it was obtained from the donor. The donors had been asymptomatic for at least 10 days, with a serum SARS-CoV-2 – specific enzyme-linked immunosorbent assay (ELISA) antibody titer higher than 1:1000 and a neutralizing antibody titer greater than 40. Patients received also anti-virals continuously until viral loads returned negative. The authors observed that fever, inflammatory markers and CT findings improved following convalescent plasma treatment. Three of 5 patients were weaned off mechanical ventilation and discharged; 2 remained hospitalized.

Ahn et al. [31] reported their experience on convalescent plasma therapy on 2 severe COVID-19 patients with ARDS in Korea. Both patients had received prior treatment with lopinavir-ritonavir and HCQ but progressed to ARDS. Both patients were commenced on methylprednisolone and convalescent plasma. Clinical, lymphocyte count and radiologic improvement, as well as viral clearance were seen. One was weaned off ventilator and the other was discharged. Neutralizing antibody titres were not assessed.

While the initial results appear to be promising, the evidence is limited by the observational nature of the studies and small sample sizes. Recently the US FDA has listed convalescent plasma as an emergency investigational new drug for patients with serious or immediately life-threatening COVID-19, pending application from the patient’s physician and FDA approval [69]. Severe disease was defined as dyspnoea, respiratory rate ≥ 30 /min, SpO_2_ ≤ 93%, partial pressure of arterial oxygen to fracture of inspired oxygen radio < 300, and/or lung infiltrates > 50% within 24 to 48 h. Life-threatening disease was defined as respiratory failure, septic shock, and/or multiple organ dysfunction or failure. Eligible plasma donors needed to have had proven history of COVID-19; complete resolution of symptoms at least 28 days before donation or complete resolution of symptoms at least 14 days before donation and negative COVID-19 tests; tested negative for human leukocyte antigen (HLA) antibodies; and had defined SARS-CoV-2 neutralizing antibody titers (eg: greater than 1:80). The potential risks of convalescent plasma transfusion include pathogen transmission, allergic transfusion reactions, transfusion-associated circulatory overload and transfusion-related acute lung injury [70].

## Others

This section covers mesenchymal stem cell therapy, which has been investigated in COVID-19 patients, but is not included in the treatment algorithm of any of the guidelines.

### Mesenchymal stem cell treatment

The interest in mesenchymal stem cell treatment lies in the immunomodulatory effects of these stem cells, which can potentially produce anti-inflammatory effects to attenuate the cytokine storm caused by a dysfunctional immune response to the SARS-CoV-2 virus. SARS-CoV-2 invades target cells via its spike proteins, which bind to angiotensin-converting enzyme (ACE)-2 receptors that are widely distributed in many types of human cells, including the alveolar type II cells in the lungs [71, 72]. However, bone marrow which produces mesenchymal cells lack ACE-2 receptors, thus making them immune to the effects of the virus.

A literature search found 2 clinical studies describing the clinical experience with mesenchymal stem cell therapy on COVID-19 patients. Leng et al. [32] performed a clinical pilot trial of mesenchymal stem cell treatment on 7 patients (1 critical illness, 4 severe illness, 2 common illness, according to NHC criteria) in China. Three patients with severe illness served as controls. The authors reported that oxygen saturations improved to ≥95% with oxygen supplementation up to 5 L/min or without in all patients within 2 to 4 days of mesenchymal stem cell transplantation. 3 patients (1 severe, 2 common) were discharged in 10 days after receiving mesenchymal stem cell treatment. No complications were noted in the treatment group. Amongst the control group, one patient died; another patient developed ARDS. The biochemistry of only the critically severe patient was presented, which demonstrated an increase in peripheral lymphocytes and reduction in inflammatory cytokines. The authors concluded that intravenous transplantation of mesenchymal stem cells was safe and effective for treatment of patients with COVID-19, especially for the patients in critically severe condition.

Liang et al. [33] reported their experience with mesenchymal stem cell therapy on a critically-ill, intubated 65-year old lady with multi-organ failure in China. Prior to mesenchymal stem cell therapy, the patient had received a cocktail of drugs including lopinavir-ritonavir, interferon-alpha inhalation, oseltamivir, traditional Chinese medicine (Xuebijing), methylprednisolone, immunoglobulin and thymosin α_1_. Allogenic human umbilical cord MSC was administrated intravenously 3 times, 3 days apart. Following the second administration, her bilirubin, C-reactive protein, liver transaminases, white blood cell, neutrophil, CD3^+^ T cell, CD4^+^ T cell, and CD8^+^ T cell counts normalized and she was decannulated. She tested negative for the virus after 8 days and was transferred out of ICU. No side effects were observed. This study was reported in a preprint open repository server.

Based on the current evidence, the small number of patients and lack of adequate controls prevents one from drawing conclusions about the benefits of mesenchymal stem cell therapy. Little is also known about the properties of the stem cells used in the studies. In addition, the potential long-term adverse effects on the immune system are unknown [73].

## Knowledge gaps and ethical issues

This review highlights 3 main issues. The first is that clinical findings from small scale studies without control groups are largely anecdotal, which may explain the disparate results across different studies. Most of the studies identified are of level 4 and 5 (19 of 30 studies) level of evidence, and of the remaining studies, most did not have control groups. Therefore these treatments should be considered experimental and ideally investigated in the setting of a clinical trial with informed consent. Clinicians also need to consider the individual risk-benefit ratio before administering investigational drugs in the off-label context.

The second issue is the lack of information on potential adverse effects of these investigational drugs in the COVID-19 patient population. Eighteen of the 30 identified studies did not note or report adverse effects. Notwithstanding, this is an inherent issue with clinical studies of small numbers, which may not uncover safety issues especially without control groups. The concern with administering investigational drugs without full awareness of its safety profile is the assumption that drugs given as compassionate use is one step “better” than standard care alone. This is simply not true. A prime example is the elderly with cardiovascular co-morbidities, who are a high-risk group for complications and mortality from COVID-19 [8, 74]. The same population is also susceptible to the ill effects of chloroquine/HCQ if not used judiciously. The clinical trial from Brazil that prematurely halted recruitment of patients into the high-dose chloroquine arm just 6 days into the trial due to complications and deaths from cardiotoxicity illustrates this point [46]. When in doubt, the control group is considered safer in terms of risk of harm compared to the investigation group as the control group receives evidence-based care [75].

Finally, this review demonstrates that multiple heterogenous trials with small sample sizes will inevitably give rise to multiple heterogenous findings that are difficult to interpret. Recognizing the need to harmonise research efforts, the WHO is coordinating an international clinical study that focuses efforts on 4 potential treatments for COVID-19, namely lopinavir-ritonavir, lopinavir-ritonavir in combination with interferon β, remdesivir and chloroquine [76]. The same 4 therapies will also be evaluated in a European clinical trial that aims to recruit 3200 patients from 8 European countries [77]. This trial is designed to be adaptive, such that ineffective investigational treatments can be quickly rejected and replaced with others as research findings unfold, which is ideal in a study performed during an outbreak.

Limitations of this systemic review include its largely descriptive nature, because the small numbers of patients investigated for each drug and heterogenous nature of the studies precluded meta-analysis of the data. Studies that had not received peer review were included in order to share potentially important preliminary findings, which may change prior to final publication. With the emergence of results from clinical trials, this systemic review will need to be revised to reflect updated findings.

## Conclusion

The global scale of the COVID-19 outbreak has brought about much interest in identifying treatments that could potentially turn the tide. However, medical professionals are bound by the time-honored dictum to first do no harm. The current evidence of adjunctive treatments in COVID-19 does not support their routine use over standard care outside clinical trials. We eagerly await the results of quality, rigorous clinical trials that may shed light on effective and safe therapies that improve outcome especially in the severe to critically ill patient population.

## Data Availability

Not applicable.

## Abbreviations

ACE: Angiotensin-converting enzyme
NARDS: Acute respiratory distress syndrome
COVID-19: Coronavirus disease
CT: Computed tomography
ECMO: Extracorporeal membrane oxygenation
ELISA: Enzyme-linked immnosorbent assay
FiO_2_: Fraction of inspired oxygen
HCQ: Hydroxychloroquine
HLA: Human leukocyte antigen
HIV: Human immunodeficiency virus
ICU: Intensive care unit
IDSA: Infectious Diseases Society of America
IL-6: Interleukin 6
LMWH: Low molecular weight heparin
MERS-CoV: Middle East respiratory syndrome coronavirus
NHC: National Health Commission
*OR*: Odds ratio
PaO_2_: Partial pressure of arterial oxygen
RCT: Randomized controlled trial
*RR*: Relative rate
SARI: Severe acute respiratory infection
SARS-CoV: Severe acute respiratory syndrome coronavirus
SIMIT: Società Italiana di Malattie Infettive e Tropicali
SWAB: Stichting Werkgroep Antibiotica Beleid
SaO_2_: Arterial oxygen
SpO_2_: Peripheral oxygen saturation
US FDA: United States Food and Drug Administration
WHO: World Health Organization
WMD: Weighted mean difference
